# Label-Free Imaging Techniques to Evaluate Metabolic Changes Caused by Toxic Liver Injury in PCLS

**DOI:** 10.3390/ijms24119195

**Published:** 2023-05-24

**Authors:** Svetlana Rodimova, Artem Mozherov, Vadim Elagin, Maria Karabut, Ilya Shchechkin, Dmitry Kozlov, Dmitry Krylov, Alena Gavrina, Nikolai Bobrov, Vladimir Zagainov, Elena Zagaynova, Daria Kuznetsova

**Affiliations:** 1 Institute of Experimental Oncology and Biomedical Technologies, Privolzhsky Research Medical University, 10/1 Minin and Pozharsky Sq., 603000 Nizhny Novgorod, Russia; srodimova123@gmail.com (S.R.);; 2 Laboratory of Molecular Genetic Research of the Institute of Clinical Medicine, Lobachevsky Nizhny Novgorod National Research State University, 23 Gagarina Ave., 603022 Nizhny Novgorod, Russia; 3 The Volga District Medical Centre of Federal Medical and Biological Agency, 14 Ilinskaya St., 603000 Nizhny Novgorod, Russia; 4 Nizhny Novgorod Regional Clinical Oncologic Dispensary, Delovaya St., 11/1, 603126 Nizhny Novgorod, Russia

**Keywords:** FLIM, precision-cut liver slices, toxic damage, liver pathology, metabolism

## Abstract

Abuse with hepatotoxic agents is a major cause of acute liver failure. The search for new criteria indicating the acute or chronic pathological processes is still a challenging issue that requires the selection of effective tools and research models. Multiphoton microscopy with second harmonic generation (SHG) and fluorescence lifetime imaging microscopy (FLIM) are modern label-free methods of optical biomedical imaging for assessing the metabolic state of hepatocytes, therefore reflecting the functional state of the liver tissue. The aim of this work was to identify characteristic changes in the metabolic state of hepatocytes in precision-cut liver slices (PCLSs) under toxic damage by some of the most common toxins: ethanol, carbon tetrachloride (CCl4) and acetaminophen (APAP), commonly known as paracetamol. We have determined characteristic optical criteria for toxic liver damage, and these turn out to be specific for each toxic agent, reflecting the underlying pathological mechanisms of toxicity. The results obtained are consistent with standard methods of molecular and morphological analysis. Thus, our approach, based on optical biomedical imaging, is effective for intravital monitoring of the state of liver tissue in the case of toxic damage or even in cases of acute liver injury.

## 1. Introduction

Currently, modern methods of multiphoton microscopy with fluorescence lifetime imaging microscopy (FLIM) and second harmonic generation (SHG) modalities are being actively implemented in biomedical research. Multiphoton excitation in the near infrared range makes it possible to obtain images with a high penetration depth of the laser radiation but with minimal photodamage to the tissues, making this method especially relevant for intravital examination [1]. Since the metabolic coenzyme NAD(P)H is autofluo-rescent, it can be monitored non-destructively and without exogenous labels using such optical techniques [2,3,4]. Second harmonic generation is a nonlinear coherent process for generating secondary electromagnetic waves of doubled frequency as a result of nonlinear interaction of the electromagnetic wave with non-centrosymmetric molecules, in particular with collagen [5,6]. Furthermore, the fluorescence is characterized not only by its intensity, but also by the fluorescence lifetime, i.e., the time a fluorescent molecule spends in an excited state before emitting a photon as it decays back to its ground state. Although the fluorescence lifetimes of various fluorophores in solutions have been established, in cells the fluorescence lifetime depends on the type of molecule, its conformation, and the way the molecule interacts with its environment [7,8]. For NAD(P)H the fluorescence lifetime has both short and long components that reflect whether the coenzyme is in its free or protein-bound state, respectively [2]. Therefore, measuring its fluorescence lifetime using FLIM allows estimation of the relative contributions of the free and bound forms of NAD(P)H [9]. The free form of NAD(P)H is localized in the cytosol and participates in glycolysis reactions, while the bound form of NAD(P)H is located in mitochondria and participates in the tricarboxylic acid cycle and in oxidative phosphorylation (OXPHOS) [10]. Using FLIM, it is possible to assess changes in the intensities of glycolysis, OXPHOS and biosynthetic processes in cells in various conditions [11]. Currently, the effectiveness of the FLIM method in analyzing the metabolic state of hepatocytes both in their normal state and in pathological condition has been confirmed by a number of authors [12,13,14,15], and by our group [16,17]. However, there is still a lack of data on the relative fluorescence lifetime contributions of various forms of NAD(P)H in liver with toxic damage.

Tissue slices are a common model for investigating pathological conditions for various purposes such as drug screening, the identification of new targets for therapy and for testing new diagnostic methods. Such a variety of tasks is possible due to the fact that the complex multicellular tissue architecture is preserved in the tissue slices, including the native components of the intercellular matter and those involved in cellular interactions [18,19]. The use of model systems based on liver slices is preferable to in vitro models (mono or mixed cultures of liver cells). Even under optimized conditions, the cultivation of primary hepatocytes is still challenging due to their limited ability to adhere and proliferate. In addition, it is known that the metabolism and phenotypes of isolated hepatocytes are significantly changed in comparison with their metabolism within tissues [20,21,22]. Another widely used approach is the use of in vivo animal models; however, the individual characteristics of each animal contribute to the development of its pathology, and this complicates interpretation of the results. Moreover, the induction of pathology based on an animal model requires a long period of time and a large number of animals in each experimental group [19]. Currently, the possibility of induction of such pathological conditions as alcoholic liver disease, non-alcoholic fatty liver disease, viral hepatitis (B and C) and toxic liver damage has been demonstrated based on precision-cut liver slices (PCLSs) [19]. The PCLS model is highly effective due to its preservation of the key events and cellular components of pathological changes, in particular the activation of fibrogenesis. In addition, the liver cells retain their metabolic state and their functionality; in particular, stellate liver cells retain the ability to activate into myofibroblasts and initiate fibrogenesis [23].

The most widely used hepatotoxic agents to induce injury are acetaminophen (APAP), a well-known toxic compound that causes necrosis of liver tissue, carbon tetrachloride (CCl4), which causes fibrosis and necrosis of hepatocytes, and ethanol, which causes tissue damage and oxidative stress [23,24,25,26].

Thus, the aim of our work was to identify specific optical parameters indicating toxic damage to the liver tissue under the action of various toxic agents using the modern label-free methods of multiphoton microscopy, SHG and FLIM.

## 2. Results and Discussion

**Figure 1:**
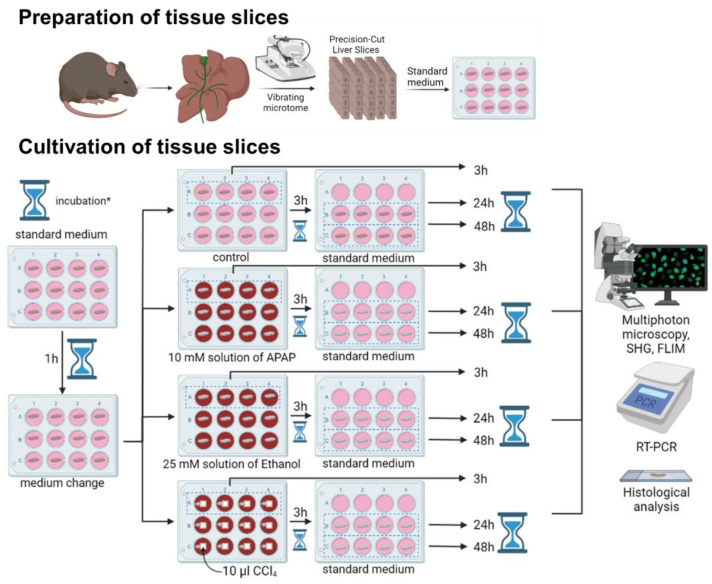
Road map of the steps in the preparation, cultivation and study of precision-cut liver slices exposed to toxins. A comprehensive analysis was performed for PCLS cultured in medium for 3 h, 24 h and 48 h; * the hourglass represents incubation.

The paper analyzes the structural and functional state of the liver tissue in toxic damage using the model of PCLSs. The design of the study is depicted in Figure 1.

### 2.1. Multiphoton Microscopy and SHG

Using multiphoton microscopy, it was shown that liver slices cultured in a standard medium (control) had a uniformly distributed NAD(P)H autofluorescence signal with a small number of bright inclusions (signal of vitamin A). In addition, SHG analysis showed there was no accumulation of collagen in the controls. On average, the NAD(P)H autofluorescence intensity values were 287.7 ± 63.6 arb. u. for 3 h, 278.1 ± 50.3 arb. u. for 24 h, and 322.4 ± 55.3 arb. u. for 48 h of cultivation.

**Figure 2:**
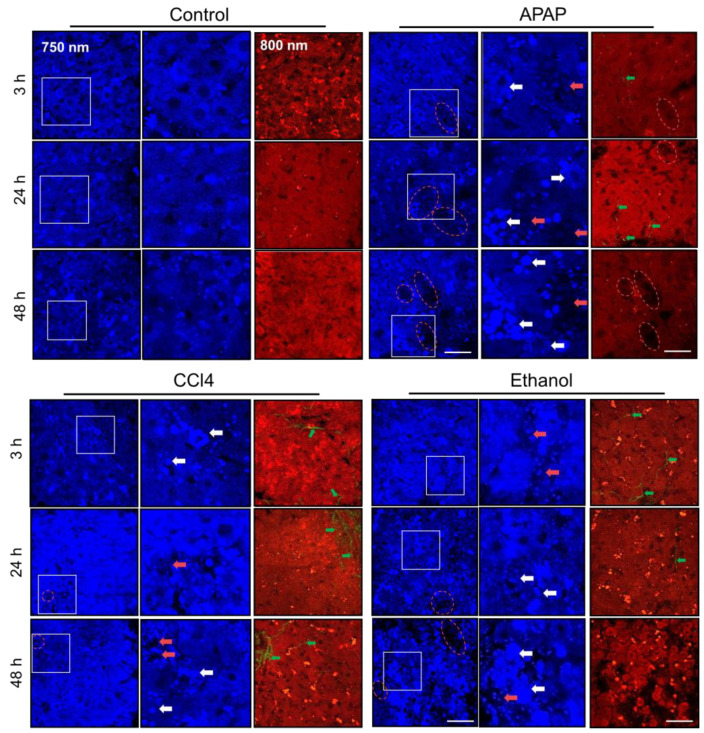
Autofluorescence microimages of liver slices exposed to toxic agents, two-photon excitation at 750 nm and 800 nm; green arrows indicate collagen, the white arrows indicate lipid droplets, the red dotted line and red arrows indicate dead cells; scale bar 50 µm.

When exposed to APAP, by 3 h of cultivation in a medium with APAP, hepatocytes with high and low NAD(P)H autofluorescence intensity were already observed in the liver slices. This was associated with lipid infiltration in the hepatocytes, which was characterized by the high intensity of NAD(P)H autofluorescence (compared to the surrounding hepatocytes with normal structure). The maximum number of such cells was observed after 48 h of cultivation (Figure 2 and Figure S2, Table S1). It is notable that even in zones with a medium intensity of NAD(P)H autofluorescence, the values were significantly higher compared to the corresponding values in the control (Table S1, Figure S2). It is known that in hepatocytes at an early phase of apoptosis, the NAD(P)H autofluorescence intensity sharply increases, while in the late phase of apoptosis, when the mitochondrial membrane potential is no longer maintained, a sharp drop in the NAD(P)H autofluorescence intensity occurs [10,27,28]. In addition, a low level of NAD(P)H autofluorescence intensity is characteristic of necrotic cells [29]. Thus, the hepatocytes with a high NAD(P)H autofluorescence intensity presumably represent an early stage of damage (early stage of apoptosis), while hepatocytes with a low intensity represent necrotic cells. Using SHG, we did not observe a significant accumulation of collagen fibers at any of the time points (Figure 2).

For the CCl4 model, at 3 h of cultivation, the hepatocytes were already characterized by a high degree of lipid infiltration (cells with a high NAD(P)H autofluorescence intensity—the initial phase of cell damage). At 24 h and 48 h of cultivation, most hepatocytes had a high intensity of NAD(P)H autofluorescence and a high degree of lipid infiltration. At the same time, no zones with a low NAD(P)H autofluorescence intensity were found (Figure 2 and Figure S2, Table S1). In addition, at 3 h of cultivation, we found a slight accumulation of collagen fibers in the tissue. In zones with medium autofluorescence intensity, the values of this parameter were the same as in the controls, except for 24 h of cultivation, where the values were significantly higher (Table S1, Figure S2). At 24 h and at 48 h of cultivation, we observed a large number of collagen fibers (Figure 2). This result is consistent with the characteristic morphological changes observed with CCl4 exposure [30,31]. Although the process of fibrosis in vivo is considered to be the result of chronic exposure, several authors have indicated early phases of fibrosis occurring as early as after just 16 h of CCl4 exposure, involving the induction of biomarkers for the activation of hepatic stellate cells, hepatocytes, endothelial cells and Kupffer cells in liver slices. In particular, using a gene expression in vivo database it has been shown that rat PCLS can imitate the toxicity and at least part of the pathology observed in vivo (such as in APAP and CCl4 models) [32].

For the ethanol model, after 3 h of cultivation, most hepatocytes were characterized by a high NAD(P)H autofluorescence intensity, which exceeded normal physiological values, indicating an acute reaction of the cells to the toxin. At 24 h and 48 h, we observed cells with low NAD(P)H autofluorescence intensity (presumably necrotic cells) (Figure 2 and Figure S2, Table S1). The number of lipid droplets in the cells gradually increased and reached a maximum by 48 h of cultivation. At the same time, the intensity values in the zones with a medium intensity of NAD(P)H autofluorescence significantly exceeded the corresponding values in the control. The accumulation of collagen fibers was negligible (Figure 2).

### 2.2. FLIM Analysis

Previously, our group has shown that by using FLIM we could determine the characteristics of the metabolic state of hepatocytes in various pathologies, such as acute cholestasis, and chronic fibrosis [16]. We also obtained FLIM data for regenerating liver [17,33]. Thus, FLIM represents a promising approach for analysis of the metabolic state of hepatocytes in the normal state and in pathological conditions; however, the field of FLIM analysis of the liver is currently at the pioneering stage. While few works have investigated the possibility of using FLIM in the study of liver tissue, there is data on the fluorescence lifetimes of free and bound forms of NAD(P)H without studying their relative contributions, and this particular approach provides information on changes in the intensity of glycolysis and OXPHOS [34]. However, there is still not enough data on the metabolic state of hepatocytes exposed to toxic damage.

An overdose of APAP increases its metabolism by CYP2E1, in turn increasing the amount of NAPQI, which is highly reactive and is primarily hepatotoxic [32,35]. Detoxification of NAPQI occurs through its binding to glutathione (GSH) to form APAP-GSH, but as the levels of GSH fall this reduces the detoxifying capacity of the liver and further contributes to cellular oxidant stress. In the absence of free GSH, any excess of NAPQI interacts with macromolecules within the hepatocytes, forming NAPQI-protein adducts, leading to mitochondrial dysfunction and to a loss of cellular ATP [36,37].

Ethanol is metabolized to acetaldehyde by oxidation, and this is rapidly metabolized to acetate, with the concurrent reduction of NAD+ to NADH. The NADH is then oxidized in the mitochondria. Acetaldehyde also has the capacity to form protein adducts in hepatocytes, and it has been proposed that these can initiate pathological processes [38,39].

When CCl4 enters an animal’s body, it is activated by the liver CYP2E1 to generate the free radicals CCl_3_·and CCl_3_O_2_· that react with oxygen to form ROS that can cause peroxidation of the fatty acids of the mitochondrial membrane and mitochondrial structure, culminating in mitochondrial dysfunction. Such mitochondrial dysfunction can lead to the uncoupling of oxidative phosphorylation from cellular respiration, resulting in the generation of additional ROS, precipitating hepatocyte degeneration and necrosis [26,40].

The mechanisms of hepatocyte damage by all three toxins we have investigated are shown in Figure S1.

Using FLIM, we analyzed the metabolic state of hepatocytes under normal conditions (without adding toxins) and under exposure to ethanol, CCl4 and APAP. The analysis of a1 and a2 provides information about the intensity of glycolysis and OXPHOS [41,42]. To a greater extent, we relied on the results of the analysis of the values of a2, since these are associated with OXPHOS, which is the main pathway for energy metabolism in hepatocytes. For all three models, the values of a2 in the control slices at 24 h and 48 h did not differ significantly. The fluorescence lifetime values of the two forms of NAD(P)H, and their relative contributions, for each control group of liver slices are presented in Tables S2 and S3.

**Figure 3:**
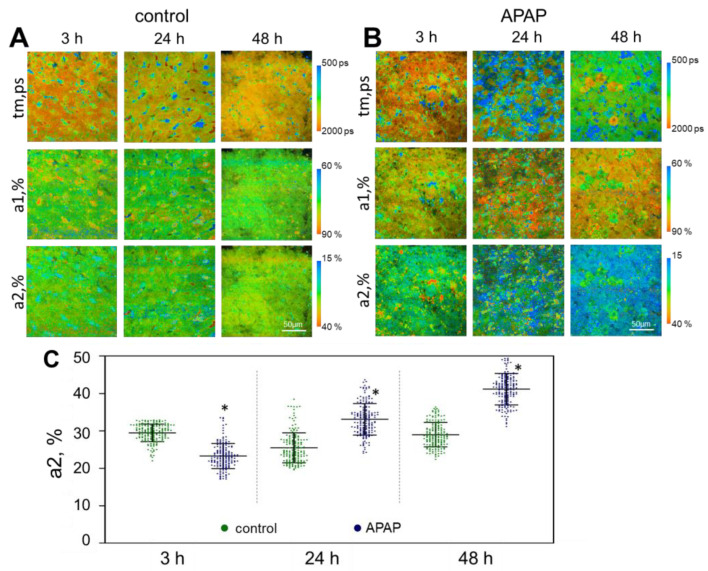
Metabolic imaging of control liver slices and those exposed to APAP. (**A**,**B**) pseudo-colored FLIM-images of liver slices; (**C**) scatter plots reflecting the distribution of the values of the fluorescence lifetime contributions of the bound form of NAD(P)H; tm (ps) is the amplitude-weighted mean lifetime (tm); a1 (%) and a2 (%) are the relative contribution of the free and bound form of NAD(P)H, respectively; *—statistically significant difference compared to the corresponding time point for control liver slices; scale bar: 50 μm.

In the APAP model, after 3 h of incubation, we could already observe a pronounced heterogeneity of hepatocytes in terms of their metabolic state due to the presence in the tissue of both metabolically active and dying cells. Dying cells could be distinguished by their large number of bright inclusions in the cytoplasm and inadequate values of FLIM parameters; therefore, such cells were not analyzed. The values of the fluorescence lifetime of the free form of NAD(P)H (t1, ps) were reduced after 24 h of cultivation; however, after 48 h of cultivation, the values increase sharply and significantly exceed the corresponding values in the control. The fluorescence lifetime values of the bound form of NAD(P)H (t2, ps) at 3 h and 24 h of cultivation were significantly reduced in comparison with the control data. The values of the amplitude-weighted mean fluorescence lifetimes of NAD(P)H (tm, ps) at 3 h and 24 h of cultivation were significantly reduced, while at 48 h there was a sharp and significant growth in the values of this parameter compared to the control data (Table S1). There was a sharp increase in the contribution of the bound form of NAD(P)H at 24 h, and this was even more pronounced at 48 h (Figure 3, Table S3). Thus, the values of a2 had significantly increased compared to the control liver slices (without toxin), which is not consistent with the common mechanism of action of APAP. Specifically, APAP overdose leads to mitochondrial dysfunction and to the disruption of the mitochondrial respiratory chain [42]. On the other hand, exposure to APAP increases the [NADH]/[NAD+] ratio in hepatocytes, although it is still not completely clear which form of NADH predominates in this process. So far, only the disruption of the mitochondrial complexes by APAP has been identified, but the data on which particular complex is damaged are rather contradictory. In a number of works, the authors show that NAPQI inhibits both NADH (complex I)- and succinate (complex II)-driven respiration in isolated mouse liver mitochondria, apparently without affecting complexes III and IV. In particular, the activities of complex I (NADH dehydrogenase), complex II (succinate dehydrogenase) and mitochondrial ATPase were significantly reduced in mice with APAP-induced liver injuries [43]. However, in other works, spectrophotometric studies showed that the NADH dehydrogenase (complex I) worked normally in the presence of APAP if it was isolated from the mitochondrial electron transport chain. This suggests that APAP affects not complex I itself, but the electron transport from complex I to complex III. In addition, the main process of cytotoxicity is the accumulation of NAPQI-adducts, since mitochondrial respiratory function is maintained even after initial adduct formation [44]. In our work, a significant increase in the contribution of the bound form of NADH confirmed the preservation of mitochondrial complex I activity. This indicates that with the accumulation of NADH in the cell, the number of NADH molecules bound to the NADH dehydrogenase of mitochondrial complex I increases and electron transport is impaired, which probably leads to the accumulation of bound forms of NADH.

**Figure 4:**
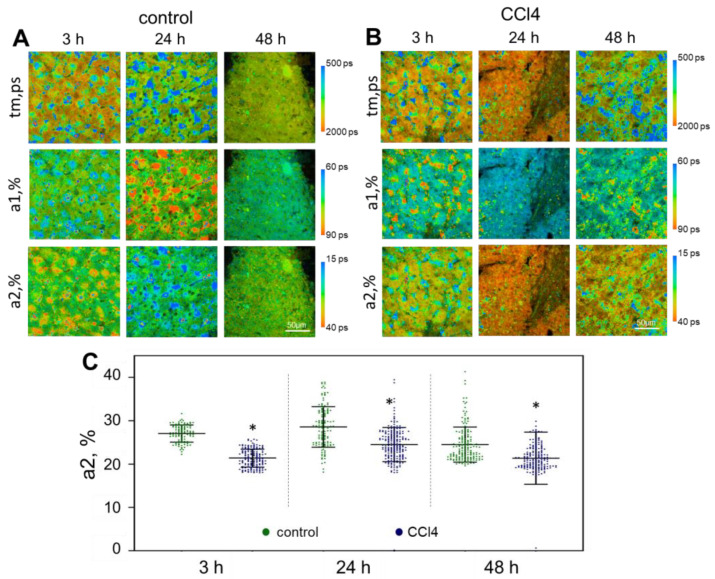
Metabolic imaging of control liver slices and those exposed to CCl4. (**A**,**B**) pseudo-colored FLIM-images of liver slices; (**C**) scatter plots reflecting the distribution of the values of the fluorescence lifetime contributions of the bound form of NAD(P)H; tm (ps) is the amplitude-weighted mean lifetime (tm); a1 (%) and a2 (%) are the relative contribution of the free and bound form of NAD(P)H, respectively; *—statistically significant difference compared to the corresponding time point for control liver slices; scale bar: 50 μm.

The CCl4 model showed the presence of cellular heterogeneity, more pronounced after 48 h, indicating a later manifestation of toxicity in this model compared to the APAP model. After incubation with CCl4, the fluorescence lifetimes of the free (t1, ps) and bound forms (t2, ps) of NAD(P)H did not differ significantly from the values in the control at any of the monitoring time points. However, the values of the amplitude-weighted mean fluorescence lifetime of NAD(P)H (tm, ps) at 24 h and 48 h of cultivation were significantly reduced in comparison with the control data (Table S2). We also observed a gradual decrease in the contribution of the bound form of NAD(P)H (a2,%) up to 48 h of cultivation (Figure 4, Table S3). The CCl4 damage is associated with mitochondrial oxidative stress, membrane potential disruption, and mitochondrial respiratory chain dysfunction [45]. As a result, the [NAD+]/[NADH] ratio is decreased, leading to impairment of mitochondrial metabolism and to excessive ROS production, which could further promote mitochondrial injury [46]. Furthermore, Hua H. et al. showed a significant reduction of mitochondrial NADH dehydrogenase 1—the core subunit of the mitochondrial membrane respiratory chain (Complex I), inducing mitochondrial dysfunction [47]. Reduction of the NADH dehydrogenase is associated with insufficient binding of the enzyme to the cofactor NADH, ultimately leading to an accumulation of the free form of NADH [48]. Such observations are consistent with our results. A decrease in the contribution of the NAD(P)H bound form is generally associated with a cellular metabolic shift to a more glycolytic state with a reduced intensity of the OXPHOS pathway. Since OXPHOS is the principal pathway that normal hepatocytes use for obtaining energy, a low a2 value indicates that the hepatocytes are suffering damage or hypoxia [49].

**Figure 5:**
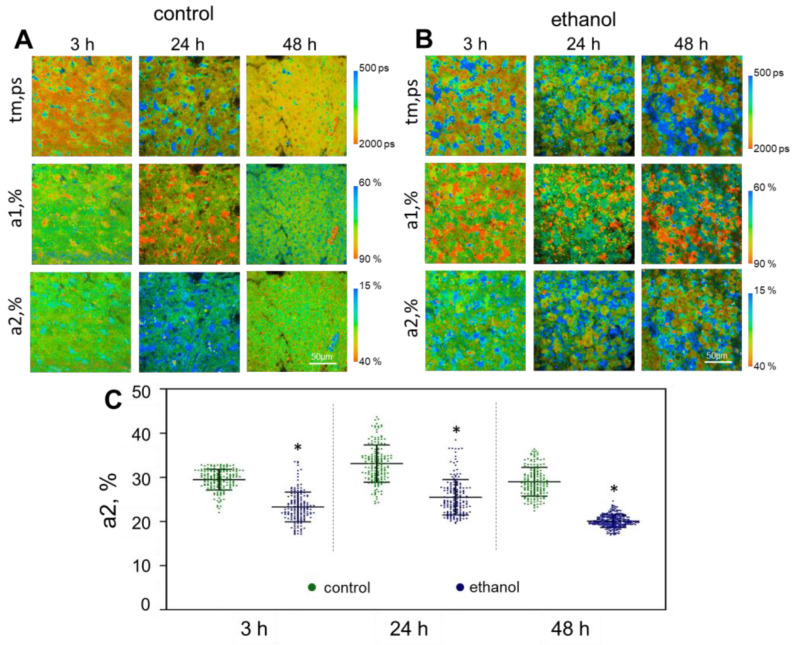
Metabolic imaging of control liver slices and those exposed to ethanol. (**A**,**B**) pseudo-colored FLIM-images of liver slices; (**C**) scatter plots reflecting the distribution of the values of the fluorescence lifetime contributions of the bound form of NAD(P)H; tm (ps) is the amplitude-weighted mean lifetime (tm); a1 (%) and a2 (%) are the relative contribution of the free and bound form of NAD(P)H, respectively; *—statistically significant difference compared to the corresponding time point for control liver slices; scale bar: 50 μm.

As in the case of the other models, the characteristic feature of the ethanol model was the presence of cell heterogeneity in terms of their metabolic state, which indicates the presence of living and necrotic (apoptotic) cells. The fluorescence lifetimes of the free (t1, ps) and bound (t2, ps) forms of NAD(P)H did not differ significantly from the values in the control at any of the monitoring time points. However, the values of the amplitude-weighted mean fluorescence lifetimes of NAD(P)H (tm, ps) at 24 h and 48 h of cultivation were significantly reduced in comparison with the control (Table S2). After 24 h of incubation, there was a slight decrease in the values of the contribution of the a2 (%). After 48 h, it had decreased significantly in comparison with the control at the same time point (Figure 5, Table S3). As in the case of APAP and CCl4 exposure, under the influence of ethanol there was a sharp increase in the ratio of [NADH]/[NAD+]. Recently, Goodman R. P. et al. have applied a genetic tool, a bacterial NADH oxidase from LbNOX, which can be targeted to different subcellular compartments. In this work, the authors used LbNOX with metabolomics to characterize the biochemical consequences of lowering the hepatic, free cytosolic [NADH]/[NAD+] ratio in vivo. The authors showed that ethanol, which generates cytosolic NADH through its oxidation by alcohol dehydrogenase, increased the lactate/pyruvate ratio, indicating a high free NADH concentration in the mitochondria, which is in complete agreement with our FLIM data [50]. A decrease in the contribution of the NAD(P)H bound form indicates a shift to a more glycolytic state with a reduced intensity of the OXPHOS pathway, which is one of the reasons for the damage to the hepatocytes [49].

### 2.3. Real-Time PCR

**Figure 6:**
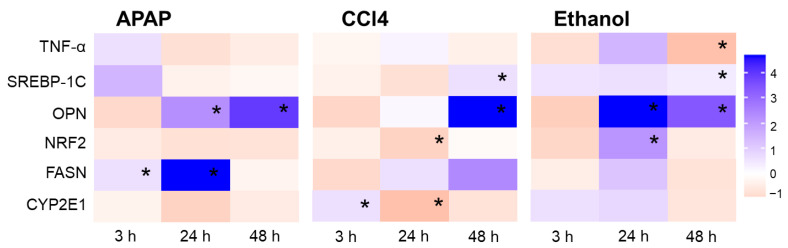
Heat map of gene expression; the color scale indicates the level of gene expression. Data are normalized for each monitoring time point relative to controls. Numerical designations on the color scale reflect by how many fold the gene expression has changed relative to the corresponding control data. *—statistically significant difference compared to the corresponding time point for control liver slices.

To confirm induced toxic damage, we assessed the level of expression of genes associated with toxic damage to liver tissues—Tnf-α, Srebp-1c, Opn, Nrf2, Fasn, and Cyp2e1 (Figure 6).

Tumor necrosis factor (TNF-α) is a cytokine that triggers pro-inflammatory effects associated with NF-κB, and apoptotic processes through caspase-8 activation. In addition, an excess of TNF-α is known to accelerate the production of triglycerides in the liver, which contributes to the development of hyperlipidemia [51]. Normally, the liver and other tissues are able to produce only minimal levels of TNF-α. However, TNF-α is mainly produced by activated Kupffer cells and is involved in the pathophysiology of alcoholic liver disease. A significant increase in the level of expression of TNF-α has also been shown in a mouse model of CCl4-induced fibrosis [51], and in a study of APAP exposure, a large number of Kupffer cells were already activated within 1 h and were secreting TNF-α, IL-6 and IL-1β [52]. In our work, at 3 h of cultivation, there was a trend towards an increase in the expression of the TNF-α in the APAP and CCl4 models, while in the ethanol model, we observed a trend towards a decrease in the TNF-α expression. After 24 h, there was a trend towards a decrease in the expression of the TNF-α in the APAP model, but an increase in expression for the ethanol and CCl4 models. After 48 h, a statistically significant decrease in the expression of the TNF-α was found for the ethanol model, while in the APAP and CCl4 models, the expression of this gene did not differ significantly from the controls at 48 h (Figure 6).

Sterol regulatory element-binding transcription factor 1 (SREBP-1c) is one member of the SREBP family that transcriptionally activates genes required for lipogenesis. SREBP-1 plays a key role in the induction of lipogenesis when mTORC1 is activated [53]. A number of works describe an increase in the level of STERP expression in response to the effects of APAP [54,55,56], ethanol [54] and CCl4 [57]. However, in our work, a pronounced increase was observed only in the early stages of cultivation with the toxins. After 48 h of cultivation with CCl4 and ethanol, the expression of SREBP-1c did significantly increase again, but the changes were not so pronounced.

The gene *opn* encodes a protein, osteopontin, which is a marker of various pathological conditions, including inflammation and fibrogenesis, and is also involved in the regulation of cell proliferation and migration [58]. In all three models of toxic damage, we observed a trend towards a decrease in OPN gene expression after 3 h of cultivation, but, by 24 h, we detected a statistically significant upregulation in gene expression in the APAP and ethanol models by 3.3 and 6 fold, respectively. For the CCl4 model, there was a trend towards an increase in gene expression, but no statistically significant differences were found compared to the control. After 48 h of cultivation, there was a statistically significant increase in the expression of OPN for all models relative to the control data (Figure 6). An upregulation of OPN correlates with the morphological and SHG analysis data, which also showed accumulation of collagen fibers in the liver slices starting from 24 h of cultivation. In the liver, hepatic Kupffer cells secrete osteopontin, facilitating the infiltration of macrophages and neutrophils into areas of necrotic liver cells when exposed to various kinds of toxins. In addition, OPN upregulation has been identified in the early stages of fibrosis [59,60].

*Nrf2* encodes a nuclear factor erythroid 2-related factor 2 (NRF2) involved in adaptive responses to oxidative stress through interaction with sequences of the antioxidant response element [61]. After 3 h of cultivation, in the APAP and ethanol models, we observed a tendency towards a decrease in the expression of NRF2. By contrast, in the CCl4 model, we revealed an increase in the expression of this gene relative to the control values after 3 h of cultivation. After 24 h, for the ethanol model, there was a most pronounced increase in the expression of NRF2 (by 2.6 fold), which is consistent with known data, since NRF2 activation in hepatocytes is primarily specific for chronic ethanol exposure [62] and the mechanism for NRF2 activation presumably relates to ethanol-induced oxidative stress [63]. In addition, there was a statistically significant decrease in the expression of this gene in the CCl4 model (by 1.6 fold). For the APAP model, we observed a trend towards a decrease in the expression of NRF2, but no statistically significant differences were found. After 48 h, there was a trend towards an increase in the expression of NRF2 for the CCl4 model, and a decrease in its expression for the APAP and ethanol models in comparison with the control data (Figure 6).

Fatty acid synthase (FASN) catalyzes the last step in fatty acid biosynthesis. FASN synthesizes long-chain fatty acids from Acetyl-CoA [64]. After 3 h of cultivation, there was a statistically significant increase in FASN expression in the APAP model, and a trend towards a decrease in expression for the CCl4 model. In the ethanol model, expression did not differ from the control after 3 h. After 24 h, for the APAP model we observed a statistically significant increase in expression (by 5.38 fold) compared with the control data. For the ethanol and CCl4 models, after 24 h there was also a trend towards increased FASN expression, but no statistically significant differences were found. After 48 h, there was a trend towards an increase in Fasn gene expression for the CCl4 model. In the ethanol and APAP models, expression did not differ from the controls at 48 h (Figure 6). Numerous works describe a decrease in the expression of FASN; in particular, FASN was downregulated in the livers of mice receiving 70 mg/kg APAP due to oxidative stress, which inhibits fatty acid synthesis [65]. However, it is also reported that upregulation of FASN in hepatocytes can be considered as a compensatory adaptation in the early stages of the development of toxic liver damage [64]. Furthermore, the level of expression of FASN has been found to increase when exposed to ethanol and CCl4, which is in complete agreement with our data [65].

CYP2E1 is a part of the mitochondrial respiratory chain and plays a role in toxin and drug metabolism. Previous studies have shown increased cellular injury, lipid peroxidation, oxidant stress and mitochondrial damage induced by ethanol, CCl4 and APAP [66,67]. In our experiments, after 3 h, a statistically significant increase in CYP2E1 expression was shown for the CCl4 model (1.7-fold) and in the ethanol model (1.4-fold), while no significant changes were found in the APAP model. After 24 h, a significant decrease in CYP2E1 expression was observed in the CCl4 model (3.3-fold), and in the APAP (1.2-fold); in the ethanol model, we observed a slight increase in expression of CYP2E1 (by 1.6 fold). In all the toxin models, after 48 h of culturing the liver slices, CYP2E1 gene expression had decreased slightly. The significant increase in the CYP2E1 expression found at the early stages in the CCl4 model is associated with the mechanisms of metabolism of this toxin, which involves the respiratory chain of the mitochondria, in particular CYP2E1 [68,69,70]. The highly destructive CCl4-derived free radicals can significantly increase the activity of CYP2E1, leading to the activation of positive feedback and finally causing severe liver damage [71,72]. With prolonged exposure to these toxins, mitochondrial dysfunction develops, which is also consistent with our data—a decrease in CYP2E1 expression at 24 h and 48 h of cultivation for all three models (Figure 6). It is probable that, by 24 h of cultivation, the reserves for maintaining high CYP2E1 activity are depleted. In the case of the APAP model, our results are in agreement with other authors—in particular, Papackova, Z., et al., who revealed that CYP2E1 activity in the liver was significantly reduced in APAP-administered mice after 6 h of exposure [73]. Gill P. et al. have also shown that CYP2E1 mRNA expression in cells treated with 5 mM APAP was down regulated by 2.2 fold at 24 h [74]. Ethanol exposure also directly and indirectly enhances the CYP2E1 activity with a resultant increase in ROS, which is consistent with our data [66,67,75].

### 2.4. Histological Analysis

**Figure 7:**
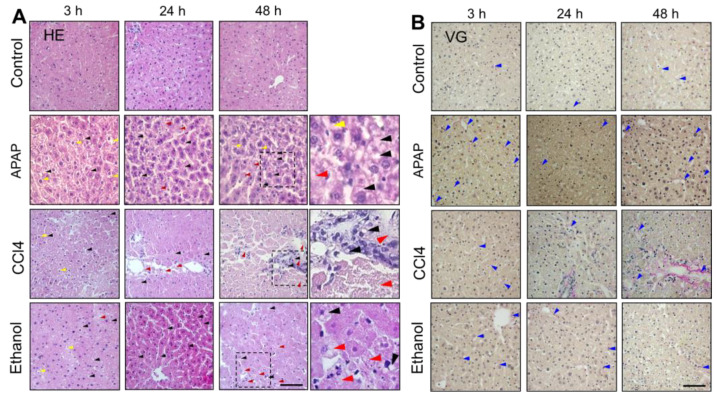
Histological images of liver tissue in the normal state and exposed to ethanol, CCl4 and APAP. (**A**) Hematoxylin and eosin (HE) and (**B**) Van Gieson (VG) staining. Black arrows indicate lipid droplets, red arrows indicate necrotic cells, yellow arrows indicate cells with edema, blue arrows indicate collagen.; ×400; scale bar: 50 μm.

Morphological analysis showed no significant dystrophic changes in liver slices cultured in the control medium at any of the monitoring time points, while the quantity of collagen fibers in the tissue was also within the normal range (Figure 7). The hepatocytes were polygonal in shape with central, rounded vesicular nuclei. The cytoplasm was acidophilic.

After 3 h of APAP exposure, we detected edema of the hepatocytes and non-significant foci of lipid infiltration (Figure 7). After 24 h of cultivation, multiple foci of lipid infiltration and individual necrotic hepatocytes could be detected (Figure 7). After 48 h of cultivation, a slight increase in the number of necrotic hepatocytes, an increase in foci of lipid infiltration in the hepatocytes and severe ballooning degeneration were shown (Figure 7). The revealed dystrophic changes are typical of the APAP toxic damage model [76].

Under the influence of CCl4, already after 3 h of cultivation, multiple foci of lipid infiltration and dystrophic changes in the hepatocytes (violation of their normal shape) were evident, but no foci of necrosis were detected. The quantity of collagen fibers was insignificant (Figure 7). After 24 h, dystrophic changes in the hepatocytes could be detected—changes in their shape and a high degree of lipid infiltration—together with a general violation of the liver tissue architecture. We also revealed extensive foci of necrosis of endothelial cells and identified a pronounced increase in the accumulation of collagen fibers (Figure 7). After 48 h of cultivation, extensive foci of necrosis of endothelial cells and significant edema in the hepatocytes was evident. The collagen accumulation had increased significantly, and a few foci of fibrosis were even observed (Figure 7). The results correspond to known dystrophic changes occurring under exposure to CCl4 [77].

After 3 h of ethanol exposure, a high degree of lipid infiltration into the hepatocytes, a significant number of hepatocytes with edema and a small number of necrotic hepatocytes were detected. We also observed an increased accumulation of collagen fibers (Figure 7). After 24 h of cultivation, the significant hepatocyte lipid infiltration of the hepatocytes and the small number of necrotic cells were still evident, but the number of collagen fibers had increased greatly even compared to 3 h of ethanol exposure (Figure 7). After 48 h of cultivation in ethanol, violation of the architecture of the liver tissue was revealed, while the number of cells with lipid infiltration had decreased sharply. The number of necrotic cells had not changed significantly compared to 24 h of cultivation in this medium. At 48 h of cultivation, we revealed edema of hepatocytes and pronounced destructive changes in them. The number of collagen fibers had increased, but even at 48 h, we did not observe any formation of foci of fibrosis (Figure 7). Although the in vivo model of ethanol toxic damage is characterized by fibrogenesis, which we did not observe in our work, we identified other major dystrophic changes characteristic of toxic liver damage caused by ethanol [78].

## 3. Materials and Methods

### 3.1. Precision Cut Liver Slices

For each model, liver slices were prepared using mice of the C57Black/6 line, weighing ~20 g. From each mouse, 30–35 liver slices were obtained.

Fresh hepatic tissue was cut into 1.0 × 1.0 cm samples. For this, a 7000 smz2 vibrating microtome (Campden Instruments Ltd., Loughborough, UK) was used to obtain the tissue slices. The samples were fixed with 10 µL of methyl cyanoacrylate glue dropped onto the specimen plates. Afterwards, the fixed samples were cut using a stainless-steel blade (7550-1-ss, Campden Instruments Ltd., Loughborough, UK), under buffered conditions with ice-cold PBS. The following slicing settings were used: frequency 80 Hz, oscillation amplitude 2 mm, sectioning speed 0.4 mm/s, with a step size of 400 µm. Immediately after cutting, the samples were pre-incubated individually in a 12-well plate and CO2-conditioned in standard culture medium consisting of Williams’ Medium E (#PM15121, Elabscience, Houston, TX, USA) supplemented with 25 mM HEPES (Gibco, Waltham, MA USA) and 10% FBS (Gibco, Waltham, MA, USA), 4 mM L-glutamine (PanEco Ltd., Moscow, Russia), 0.1 μM dexamethasone (StemCells Technologies, Waltham, MA, USA), and an antibiotic-antimycotic consisting of 100 unit/mL of penicillin, 100 μg/mL of streptomycin and 25 μg/mL of Fungizone™ (Gibco, Waltham, MA USA) for 1 h. Next, to induce APAP toxic damage, the liver slices were placed for 3 h in a 10 mM solution of APAP diluted in standard culture medium. To induce ethanol toxic damage, liver slices were placed for 3 h in 25 mM ethanol diluted in standard culture medium [19,79]. For the CCl4 model, the liver slices were incubated in 12-well plates with 2 mL standard culture medium, and a piece of filter paper soaked in 10 µL of CCl4 was attached to the lid of the 12-well plates. The incubation with CCl4 was also carried out for 3 h [19,79,80]. As a control, we used liver slices cultivated in the standard culture medium without toxins. For each model, we used a corresponding control obtained from the same animal to reduce the influence of the individual characteristics of the animal. Cultivation was carried out at 37 °C on an orbital shaker (90 rpm).

After 3 h of cultivation with toxin, a third of each sample and a third of each control was taken for immediate study while the rest was placed in standard medium for either 24 or 48 h [81]. After either 24 h or 48 h of further cultivation, all remaining liver slices (both the controls and those exposed to toxin) were taken for study. The design of the study is depicted in Figure 1.

### 3.2. Multiphoton Microscopy

Investigation of liver slices (*n* = 6–9 for each monitoring time point) was performed using an LSM 880 (Carl Zeiss, Oberkochen, Germany) equipped with a Ti:Sapphire femtosecond laser (repetition rate: 80 MHz, pulse duration less than 100 fs) and a time-correlated single photon counting (TCSPC) system (Simple-Tau 152, Becker & Hickl GmbH, Berlin, Germany). The average laser power used was about 6 mW. A C Plan-Apochromat 40x/1.3 oil immersion objective was used to collect the fluorescence signal. From 10 fields of view for each sample, both the NAD(P)H fluorescence intensity images and the FLIM data were acquired. 

To visualize the NAD(P)H fluorescence, the sample was excited at a wavelength of 750 nm, and the fluorescence signal was collected in the range 450–490 nm. At a wavelength of 800 nm, we visualized cell autofluorescence (red), detection range 433–660 nm, and SHG of collagen (green), detection range 371–421 nm. Fluorescence intensity images 1024 × 1024 pixels and 212 × 212 µm in size were obtained for visual assessment of the sample structure. We performed a quantitative assessment of the NAD(P)H autofluorescence intensity in the cytoplasm of the hepatocytes by manual selection of ~40 × 40-pixel zones as regions of interest (ROIs), using ImageJ software (version 1.43u, National Institutes of Health, Bethesda, Maryland, USA). All analyzed hepatocytes were divided into three groups, with low (<250 arb. u.), medium (250–400 arb. u.) and high (>400 arb. u.) intensity of NAD(P)H autofluorescence. The ranges of NAD(P)H autofluorescence intensity values are based on reference values for liver slices cultured in standard medium and on our previous work [17]. In each group, 10–30 measurements were taken for each image, depending on the number of cells corresponding to the analyzed group.

FLIM images of 512 × 512 pixels were acquired from the same fields of view. The FLIM analysis was performed with SPCImage software (version 8.3, Becker & Hickl GmbH, Berlin, Germany) using a bi-exponential decay model. To maintain a minimum of 5000 counts per pixel, the binning parameter was set at 2. The goodness-of-fit model was assessed by the χ2 value. The following parameters were analyzed in 20–30 regions of cell cytoplasm for each field of view: tm (ps), the amplitude-weighted mean fluorescence lifetime; t1 (ps), the fluorescence lifetimes of the free form of NAD(P)H; t2 (ps), the fluorescence lifetimes of the bound form of NAD(P)H; and the relative contributions of the free, a1 (%), and the bound, a2 (%), forms of NAD(P)H.

### 3.3. Real-Time PCR

RT-PCR analysis was performed on *n* = 5 liver slices for each monitoring time point. Isolation of total RNA from the samples was performed according to the protocol for the Quick-Direct-zol DNA/RNA Miniprep kit (#D7001, Zymo Research, Irvine, CA, USA). Before the reverse transcription reaction, the samples were treated with DNAase. Real-time PCR was performed on a Bio-Rad CFX96 machine (Bio-Rad, Hercules, CA, USA) using a reaction mixture based on SYBR Green (#7567). The PCR reaction mixture contained 1x GeneAmp PCR Buffer I (#8080129, Applied Biosystems, Waltham, MA, USA), 250 µM of each dNTP, 0.5 nM of each primer, and 1 U of Taq M polymerase (#751-100, Intifica, Saint Petersburg, Russia); the total concentration of Mg^2+^ in the reaction was 3 mM, the reaction volume was 20 µL. The temperature profiles of the cycles were: (1) 95 °C for 10 min (enzyme activation stage); (2) 35 cycles at 95 °C for 15 s, at 60 °C for 30 s and at 72 °C for 30 s; (3) hybridization at 95 °C for 1 min, and at 40 °C for 1 min; (4) analysis of the melting curves with measurements between 60 °C and 95 °C. The efficiency of the reaction was evaluated by a calibration curve method. Quantitative analysis of RT-PCR was performed using CFX Maestro 2.3 software. Gene expression analysis was performed using the delta-delta-Ct method modified by Hellemans et al. [82] to correct for amplification efficiency and normalize to multiple reference genes. The stability of reference gene expression was assessed using the integrated geNorm algorithm. The primer sequences for RT-PCR are presented in Table S4.

### 3.4. Histological Analysis

For histological studies, the liver slices (*n* = 6–9 for each monitoring time point) were fixed in a 4% solution of paraformaldehyde, passed through isopropyl alcohol and embedded in paraffin. Deparaffinized 7 µm sections were stained with hematoxylin and eosin and by Van Gieson’s method according to the standard protocol [83]. For each sample, ten microimages (×40) were obtained using a Leica DM 2500 microscope (Wetzlar, Germany). Afterwards, a standard morphological analysis of the liver tissue structure was performed, evaluating the presence of dystrophic changes in the cytoplasm of the hepatocytes, such as edema, the presence of necrotic cells, the accumulation of collagen and fatty infiltration.

### 3.5. Statistics

For each model and each time point of the experiment, we obtained 8–10 NAD(P)H autofluorescence and FLIM images. For each image, we performed a quantitative assessment of the NAD(P)H autofluorescence intensity in the cytoplasm of 30 hepatocytes (in zones with a high NAD(P)H autofluorescence intensity) and for 30 hepatocytes (in zones with a low NAD(P)H autofluorescence intensity), excluding the nuclei. For each image, we also determined the FLIM parameters in the cytoplasm of 30 hepatocytes (in zones with high NAD(P)H autofluorescence intensity). R-language was used for the statistical calculations. Statistical differences between the different groups were analyzed using the pairwise multiple comparison procedure. Differences in mean values between groups were considered significant when they were larger than might be expected by chance. After the tests for normality (Shapiro-Wilkes) and equal variance (F-test) showed a normal distribution of data, the pairwise *t*-test method was used, for pairwise comparison, using the Bonferroni correction; *p*-value ≤ 0.05.

Heat maps of gene expression were formed in R-studio (Posit, 250 Northern Ave, Boston, MA 02210, USA) using ggplot2. Gene expression data were standardized using z-score standardization to display no change as zero, decrease as below zero, and increase as above zero. After the tests for normality (Shapiro-Wilkes), the pairwise *t*-test method was used for pairwise comparison, using the Bonferroni correction; *p*-value ≤ 0.05.

## 4. Conclusions

In this work, using multiphoton microscopy, SHG and FLIM we determined specific optical criteria indicating toxic liver damage under the action of various toxic agents. The PCLSs model accurately reflects the mechanisms of toxic damage by various agents, due to its ability to preserve the complex intercellular interactions of the liver tissue. Interestingly, APAP-induced toxic damage was characterized by an increase in the contribution of the bound form of NAD(P)H, while exposure to ethanol and CCl4 showed a significant decrease in the contribution of the bound form of NAD(P)H, which reflects differences in the mechanisms of damage by each toxic agent. The results obtained by label-free multiphoton microscopy and FLIM are consistent with the data from standard methods of molecular and morphological analysis.

## Data Availability

All relevant data are included within the paper and its additional file.

## Supplementary Materials

The following supporting information can be downloaded at: https://www.mdpi.com/article/10.3390/ijms24119195/s1. Additional file S1 (presented as a PDF) includes the primer sequences for RT-PCR and extended results of FLIM analysis.

## Abbreviations

ADH: alcohol dehydrogenase; APAP: acetaminophen; CCl4: carbon tetrachloride; FASN: fatty acid synthase; FLIM: fluorescence lifetime imaging microscopy; GSH: glutathione; NADH: nicotinamide adenine dinucleotide; NADPH: nicotinamide adenine dinucleotide phosphate; NAPQI: N-acetyl-p-benzoquinone imine; NRF2: nuclear factor erythroid 2-related factor 2; OPN: osteopontine; OXPHOS: oxidative phosphorylation; PCLSs: precision-cut liver slices; ROS: reactive oxygen species; SHG: second harmonic generation; SREBP-1c: sterol regulatory element-binding transcription factor 1; TNF-α: tumor necrosis factor.
