# Supplementary material for: Label-Free Imaging Techniques to Evaluate Metabolic Changes Caused by Toxic Liver Injury in PCLS
Source: Int J Mol Sci. 2023 May 24;24(11):9195. doi: 10.3390/ijms24119195 (PMC10252319; doi:10.3390/ijms24119195)
Supplement: Supplementary file 1 [file ijms-24-09195-s001.zip › ijms-2367805-supplementary.pdf]

Supplementary Materials

Label-Free Imaging Techniques to Evaluate Metabolic Changes Caused by Toxic Liver Injury in PCLS

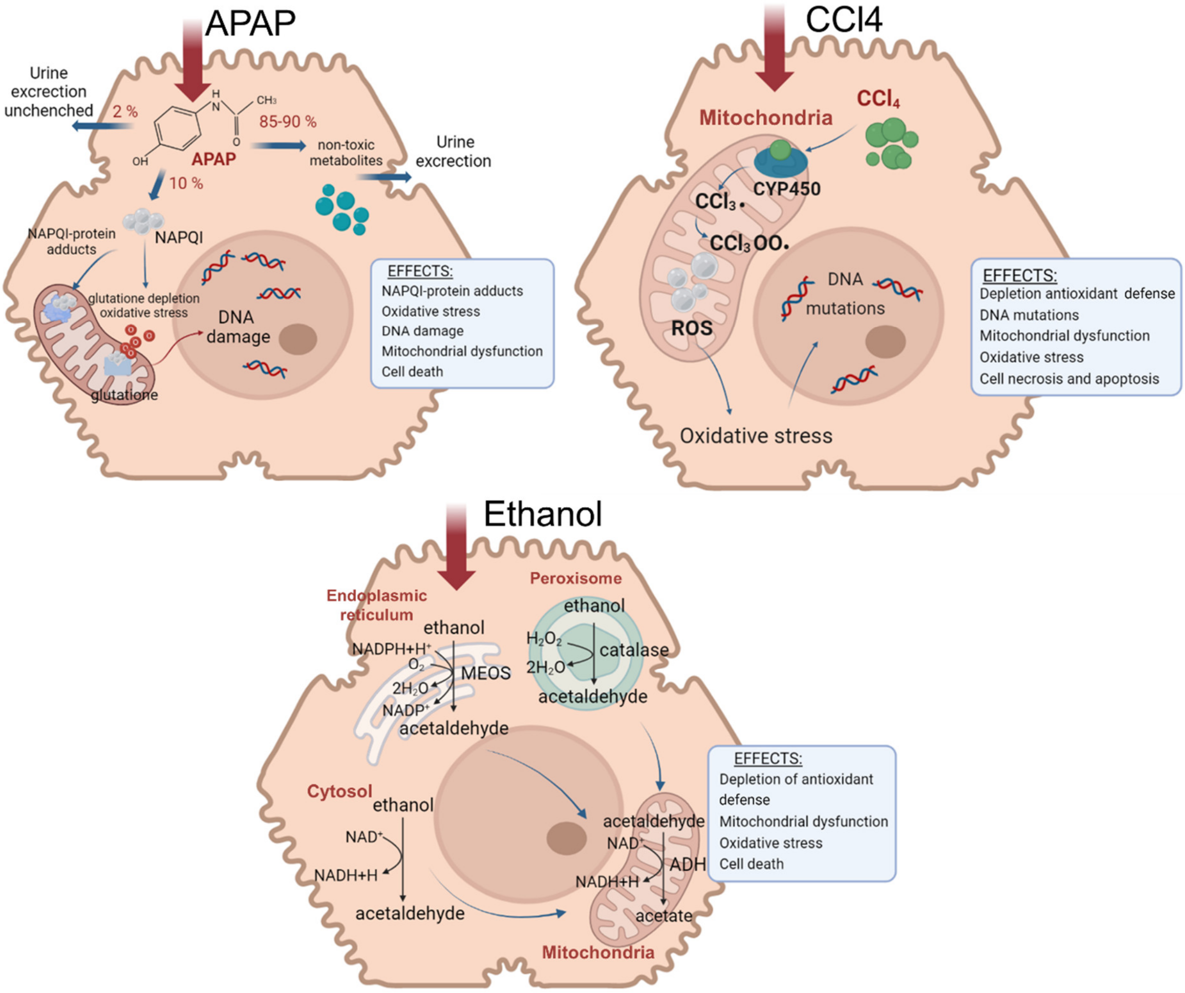

Figure S1. Schematic diagrams of the hepatotoxicity mechanisms of APAP, CCl4 and ethanol.

Table S1. Quantitative analysis of NAD(P)H autofluorescence intensity in hepatocytes of hepatic slices under toxin exposure

		Low Intencity (arb. u.)	Medium Intencity (arb. u.)	High Intencity (arb. u.)
control	3 h	-	287.7 ± 63.6	-
	24 h	-	278.1 ± 50.3	-
	48 h	-	322.4 ± 55.3	-
ethanol	3 h	210.8 ±34.7	345.7 ± 38	501.8 ± 47.7
	24 h	189 ±46.3	306.1 ± 44.7	477.5 ± 42.4
	48 h	-	359.6 ± 59.4	514.8 ± 67
CCl4	3 h	-	255 ± 49.8	454.2 ± 59.5
	24 h	-	341 ± 59.9	580.6 ± 64.4
	48 h	-	329.3 ± 48.4	569.1 ± 47
APAP	3 h	160.6 ± 32.6	358.9 ± 42.8	541.4 ± 53.8
	24 h	186.3 ± 35.9	374.5 ± 58.4	529.7 ± 36.6
	48 h	173.9 ± 41.4	348.2 ± 46.2	569.1 ± 44.7

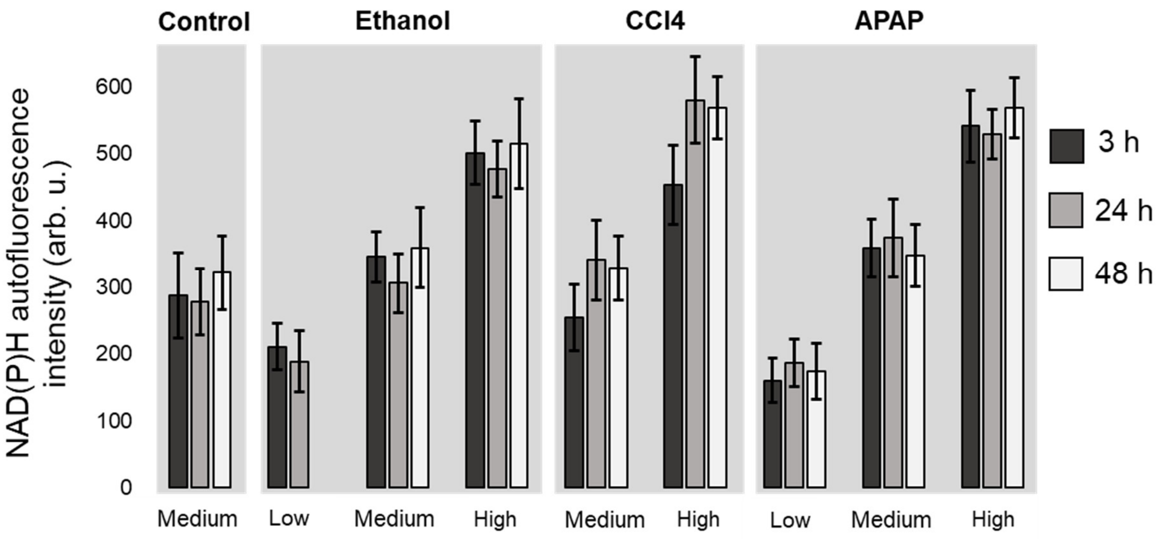

Figure S2. Quantitative analysis of NAD(P)H autofluorescence intensity in hepatocytes of hepatic slices under toxin exposure; low – low intensity of NAD(P)H autofluorescence, medium – medium intensity of NAD(P)H autofluorescence, high – high intensity of NAD(P)H autofluorescence.

Table S2. Fluorescence lifetime of free and bound form of NAD(P)H in hepatocytes of hepatic slices under toxin exposure

		t1, ps	t2, ps	tm, ps
Control	3 h	308 ± 35	2538 ± 163	917 ± 98
	24 h	308 ± 34	2290 ± 135	815 ± 111
	48 h	350 ± 15	2177 ± 201	852 ± 36
APAP	3 h	272 ± 44	1890 ± 168*	678 ± 73*
	24 h	210 ± 18*	1109 ± 104*	537 ± 90*
	48 h	444 ± 33*	2073 ± 167	1120 ± 106*

		t1, ps	t2, ps	tm, ps
Control	3 h	435 ± 32	2473 ± 116	985 ± 43
	24 h	355 ± 33	2442 ± 144	944 ± 119
	48 h	309 ±38	2497 ± 232	855 ± 123
CCl4	3 h	396 ± 39	2404 ± 110	837 ± 63
	24 h	293 ± 21	2259 ± 115	718 ± 102*
	48 h	274 ± 22	1886 ± 159*	425 ± 187*

		t1, ps	t2, ps	tm, ps
Control	3 h	354 ± 39	2427 ± 184	946 ± 195
	24 h	310 ± 43	2506 ± 191	983 ± 274
	48 h	302 ± 32	2332 ± 121	788 ± 131
Ethanol	3 h	391 ± 43	2416 ± 143	896 ± 113
	24 h	278 ± 40	2176 ± 152	713 ± 103*
	48 h	291 ± 24	2121 ± 201	659 ± 62*

*—statistically significant difference compared to the corresponding time point for control liver slices.

Table S3. Fluorescence lifetime relative contributions of free and bound form of NAD(P)H in hepatocytes of hepatic slices under toxin exposure

		a1, %	a2, %
Control	3 h	72,16 ± 2,21	27,84 ± 2,21
	24 h	69,04 ± 4,92	30,96 ± 4,92
	48 h	73,50 ± 2,13	26,50 ±2,13
Ethanol	3 h	76,85 ± 2,73*	23,15 ± 2,73*
	24 h	75,50 ± 2,63*	25,50 ± 2,63*
	48 h	79,97 ± 1,61*	20,03 ± 1,61*
		**a1, %**	**a2, %**
Control	3 h	72,95 ± 1,98	27,05 ± 1,98
	24 h	72,21 ± 4,06	27,79 ± 4,06
	48 h	75,98 ± 3,10	24,02 ± 3,10
CCl4	3 h	78,06 ± 2,06*	21,94 ± 2,06*
	24 h	77,54 ± 1,90*	22,46 ± 2,90*
	48 h	79,86 ± 3,89*	20,14 ± 3,89*
		**a1, %**	**a2, %**
Control	3 h	72,74 ± 2,06	27,26 ± 2,06
	24 h	74,46 ± 2,29	25,54 ± 2,29
	48 h	72,22 ± 2,10	27,78 ± 2,10
APAP	3 h	74,46 ± 2,29*	25,54 ± 2,29*
	24 h	66,33 ± 3,43*	33,67 ± 3,43*
	48 h	58,48 ± 3,68*	41,52 ± 3,68*

*—statistically significant difference compared to the corresponding time point for control liver slices.

Table S4. The primer sequences for RT-PCR.

Primer Target	Primer Sequence (5′→3′)
ACT-b	F: CACTGTCGAGTCGCGTCC<br>R: TCATCCATGGCGAACTGGTG
ABL1	F: TGAGCAGAAAGATGCGCCTGAC<br>R: CGCTCATCTTCATTTAGGCTGCC
SREBP-1c	F: CGACTACATCCGCTTCTTGCAG<br>R: CCTCCATAGACACATCTGTGCC
CYP2E1	F: AGGCTGTCAAGGAGGTGCTACT<br>R: AAAACCTCCGCACGTCCTTCCA
FASN	F: CACAGTGCTCAAAGGACATGCC<br>R: CACCAGGTGTAGTGCCTTCCTC
OPN	F: CTCCATCGTCATCATCATCGT<br>R: GCACCCAGATCCTATAGCC
TNF-α	F: GGTGCCTATGTCTCAGCCTCTT<br>R: GCCATAGAACTGATGAGAGGGAG
NRF2	F: ACTACAGTCCCAGCAGAGTGAT<br>R: TCACACACTTTCTGCGTGCT
